# *Aspergillus fumigatus* in the Food Production Chain and Azole Resistance: A Growing Concern for Consumers

**DOI:** 10.3390/jof11040252

**Published:** 2025-03-26

**Authors:** Katherin Castro-Ríos, Maria Clara Shiroma Buri, Arla Daniela Ramalho da Cruz, Paulo Cezar Ceresini

**Affiliations:** 1 Department of Crop Protection, Agricultural Engineering and Soil, São Paulo State University—UNESP, Ilha Solteira 15385-000, SP, Brazil; kathecas@uis.edu.co (K.C.-R.); clara.shiroma@unesp.br (M.C.S.B.); arla.daniela@unesp.br (A.D.R.d.C.); 2 Escuela de Nutrición y Dietética, Facultad de Salud, Universidad Industrial de Santander (UIS), Bucaramanga 680001, Santander, Colombia

**Keywords:** fungicide, fungistatic, azole drugs, food safety, post harvest, food contamination

## Abstract

Aspergillosis is a fungal disease caused by the inhalation of *Aspergillus* spores, with *Aspergillus fumigatus* being the primary causative agent. This thermotolerant fungus affects both immunocompetent and immunocompromised individuals, posing a significant public health concern. In recent years, the detection of *A. fumigatus* in food products and production environments has raised questions about its potential role as an additional route of exposure. Furthermore, the emergence of azole-resistant strains in agricultural settings highlights the need to better understand its transmission dynamics and implications for food safety. This review explores the occurrence of *A. fumigatus* in crops and food products, its possible routes of contamination, and the potential link between environmental exposure to azole fungicides and resistance development. Additionally, it identifies knowledge gaps and proposes future research directions to improve risk assessment and mitigation strategies within the food production chain.

## 1. Introduction

*Aspergillus fumigatus* is a widely distributed environmental fungus commonly found in soil, plant debris, and decomposing organic matter. It can also be detected in raw materials used for the production of food and beverage, and various indoor and outdoor environments [1,2,3]. The adaptability of *A. fumigatus* is attributed to its ability to grow under diverse environmental conditions, including tropical climates and varying humidity levels [4]. In general, *Aspergillus* spp. thrive at temperatures above 37 °C, while only a few can grow below 10 °C, allowing them to colonize environments ranging from tropical soils and vegetation to stored food products and indoor spaces [1,2,3].

Due to their small size, the conidia of *Aspergillus* are easily dispersed through the air, making inhalation the primary route of exposure for humans [4]. This airborne dispersal capability enhances the likelihood of fungal contamination at different stages of the food production and supply chain. *A. fumigatus* has also been detected in agricultural environments, including air, gardens, compost, plant debris, seeds, soil, water, and commercial products [5]. The use of azoles in agriculture to control fungal diseases has created an additional route for environmental exposure, increasing the risk of developing azole-resistant strains that can spread to the food chain [6].

*A. fumigatus* is the primary causative agent of aspergillosis, a group of diseases that primarily affect the respiratory system but can also impact other organs, particularly in immunocompromised individuals [5]. The severity of aspergillosis symptoms depends on the patient’s immune status and the number of spores inhaled. The disease presents in three main forms: the allergic form, which includes allergic bronchopulmonary aspergillosis (ABPA) and allergic fungal rhinosinusitis; chronic pulmonary aspergillosis (CPA), which includes chronic inflammatory forms of aspergillosis such as aspergilloma, which mainly affects patients with tuberculosis or other chronic lung diseases, and invasive aspergillosis (IA), the most severe and deadly form, affecting around 5 million people each year [5,7,8].

The opportunistic nature of *A. fumigatus* makes it particularly dangerous for individuals undergoing immunosuppressive therapies, such as chemotherapy or bone marrow transplantation, and those receiving high-dose corticosteroids [4,5]. Furthermore, the COVID-19 pandemic has highlighted the increased risk of pulmonary aspergillosis in patients requiring non-invasive ventilation, intubation, or long-term corticosteroid treatment, further emphasizing the importance of monitoring this fungal pathogen [8,9,10]. *A. fumigatus* has also recently been included by the World Health Organization (WHO), along with three other fungi, in the critical priority group of “pathogenic fungi for research, development, and public health action”; this decision was made after considering the number of deaths caused, treatment options, diagnostic access, annual incidence, complications and sequelae, and specially azole fungicide resistance [11].

Beyond its clinical significance, *A. fumigatus* produces secondary metabolites such as gliotoxin, a highly immunosuppressive substance with genotoxic, cytotoxic, and apoptotic effects [12]. Exposure to moldy agricultural products contaminated with *A. fumigatus* has been associated with neurological disorders in farmworkers, underscoring the occupational health risks associated with this fungus.

The emergence of azole-resistant strains of *A. fumigatus* is a growing public health concern, driven primarily by the widespread use of azole fungicides in agricultural settings [11]. Azoles are the primary class of antifungal agents used to treat aspergillosis, but their environmental application has led to the selection of resistant fungal strains, which can subsequently infect humans and animals [5]. Azole resistance in *A. fumigatus* has been linked to two main pathways: the clinical route, resulting from prolonged antifungal treatment in patients, and the environmental route, associated with exposure to azole fungicides in agricultural and animal husbandry settings. The increasing detection of resistant strains in farm environments raises concerns about their potential role in the emergence and spread of resistance [13,14,15,16]. However, more evidence is needed to determine the extent and mechanisms of this association.

Resistant isolates have been detected in various agricultural environments, including air, gardens, compost, plant debris, plants, seeds, soil, water, and commercial products, highlighting the role of environmental reservoirs in the spread of resistance [5]. The increasing detection of resistant strains in food production environments raises the possibility that contaminated food products could serve as an additional route for azole-resistant *A. fumigatus* exposure. However, the extent to which this environmental presence contributes to resistance development in clinical settings remains unclear and requires further investigation [6]. Recently, an isolate of *Aspergillus* sp. belonging to section *Fumigati* with Posaconazole resistance was discovered in tea samples from China [6]; this finding could represent an additional link in the environmental pathway of azole resistance acquisition by *A. fumigatus* and a direct risk for consumers. While the presence of *A. fumigatus* in food products and its increasing resistance to azoles raise public health concerns, there is currently limited direct evidence linking the consumption of contaminated food to Aspergillosis development in humans. However, given the thermotolerant nature of *A. fumigatus* and its ability to survive food processing conditions, this potential route of exposure cannot be disregarded. Further research is needed to determine whether ingestion of azole-resistant *A. fumigatus* contributes to colonization or infection, particularly in immunocompromised individuals.

The development of azole resistance is primarily linked to mutations in the Cyp51A gene, which encodes a target enzyme for azole antifungals. Environmental isolates often exhibit tandem repeat (TR) mutations combined with point mutations, such as TR34/L98H and TR46/Y121F/T289A, conferring high levels of resistance to medical azoles [5,6]. This cross-resistance between agricultural and clinical azoles jeopardizes the efficacy of frontline antifungal treatments, making the control of resistant *A. fumigatus* strains a global priority [11].

This review aims to provide a comprehensive analysis of the presence of *A. fumigatus* in crops and food products, identifying the primary sources of contamination and the possible routes by which this fungus reaches the food chain. It also examines the growing concern of azole resistance in agricultural products and its implications for food safety and public health. By addressing current knowledge gaps and proposing future research directions, this review seeks to improve risk assessment and mitigation strategies within the food production chain. Furthermore, the review explores the environmental and clinical pathways leading to azole resistance, emphasizing the importance of interdisciplinary approaches to monitor and control *A. fumigatus* contamination and resistance development.

## 2. Contamination by *Aspergillus fumigatus* in Agricultural Crops and Food Products: Sources and Risk Factors

The environmental route of azole resistance by *A. fumigatus* is related to the use of agricultural fungicides; therefore, many of the resistant isolates have been found in agricultural samples (Table 1). However, contamination with *Aspergillus* spp. could affect the entire food chain, extending from post-harvest to food processing, putting susceptible consumers at risk. A study conducted by Molinero et al. [17], which sampled isolates of *A. fumigatus* and other *aspergilli* strains from soils, compost, corn, animal feedstuffs, and soybean and chickpea seeds, seeking evidence of resistance to the main agricultural triazoles (cyproconazole, epoxiconazole, prothioconazole, and tebuconazole) and to clinical triazoles (itraconazole. voriconazole, posaconazole, and isavuconazole). No azole-resistant *A. fumigatus* isolates were found; nevertheless, other *Aspergillus* spp. showed reduced susceptibility to clinical triazoles. So, they still pose a potential risk since these species are less common causes of aspergillosis.

Currently, only one published study by Viegas et al. [30] has directly evaluated a food of direct consumption, which evidences the presence of an isolate of *Aspergillus* sp. belonging to section *Fumigati*, with resistance to posaconazole, which was obtained from tea samples from China. Regarding food industry environments, this same research group conducted a study evaluating the presence of azole-resistant fungi in ten bakeries in Portugal, using different sampling methods, finding azole-resistant *Aspergillus* spp. in environmental and raw material samples, showing a risk, especially for bakery workers [31].

These works show the possibility that *A. fumigatus* azole-resistant can spread to food for direct consumption, putting at risk the health of consumers and workers, being essential constant surveillance within food environments to detect the presence of azole-resistant isolates; however, more research is urgently needed.

### 2.1. Presence of Aspergillus fumigatus in Food Products

Fungi of the *Aspergillus* genus are common in the environment and can be found in raw materials used to produce food and beverages. Contamination by these fungi can occur at different stages of food production, including both the pre-harvest and post-harvest phases. In the field, *A. fumigatus* may colonize crops through soil, plant debris, and air dispersal; post-harvest stages, such as drying, fermentation, and storage, provide additional opportunities for fungal proliferation, particularly under conditions of high humidity and temperature fluctuations. It is essential to distinguish between contamination occurring during crop growth and that arising from improper storage practices, as these factors influence both fungal prevalence and the potential presence of azole residues

**Figure 1:**
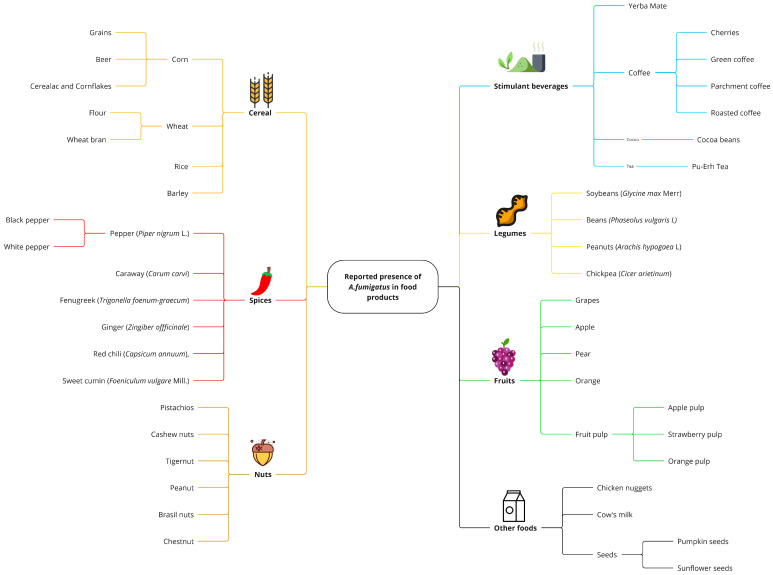
Reported presence of *A. fumigatus* in food products. Some foods may have higher contamination risks due to storage conditions or processing methods. Data are based on published studies [30,31,32,33,34,35,36,37,38,39,40,41,42,43,44,45,46,47,48,49,50,51,52,53,54,55,56,57,58,59,60,61,62,63,64,65,66,67,68,69,70,71,72,73,74,75,76,77,78,79,80,81,82,83,84,85,86,87,88,89,90,91].

*A. fumigatus* has been reported in various types of food as part of its natural environmental presence; however, its detection does not always imply contamination. Given this, it is essential to distinguish that “detection rate” refers to the frequency with which *A. fumigatus* was identified in food samples. In contrast “contamination rate” applies to cases where detection levels exceed expected environmental baselines or indicate a direct risk to food safety. A summary of the presence of *A. fumigatus* in food can be seen in Figure 1.

#### 2.1.1. Cereals

Cereals such as wheat, rice, and maize are among the most frequently reported crops contaminated by *A. fumigatus*. The presence of fungal spores in stored grains poses potential inhalation risks for workers during handling and processing [92]. Additionally, improper storage conditions, such as high humidity and temperature fluctuations, can promote fungal proliferation, increasing the likelihood of food contamination [33].

Njobeh et al. [34] evaluated the contamination in different food samples (mainly cereals and legumes), establishing that 96% of the contamination in the samples corresponded to *Penicillium* spp. and *Aspergillus* spp.; meanwhile, *A. fumigatus* was reported in 19 of the 95 samples evaluated, but its presence in cereals was evident only in corn, probably related to the high humidity conditions in harvest for this cereal. Something similar was evidenced by Sandona et al. [35] in 2019, who noticed that conditions such as high temperatures, high humidity, and the presence of organic matter (corn) favor the presence of thermotolerant and thermophilic fungi, which include *A. fumigatus*, that were isolated and identified in the samples evaluated (7% of the isolates). In another study carried out in South Africa, 100 samples of corn from markets and silos were evaluated, finding a frequency of contamination in the samples mainly of the genera *Fusarium*, *Penicillium*, and *Aspergillus*; among these, *A. fumigatus* was the most predominant species with 27%; the growth of this type of fungi is attributed to storage conditions such as variations in humidity and temperature, which promote the growth [36]. On the contrary, in a study conducted by Ekpakpale et al. [37], there was no evidence of contamination with *A. fumigatus* in cereals like corn and rice, although it is noteworthy that 81% of the samples showed contamination by *Aspergillus* spp.; the fungus *A. fumigatus* was only found in cassava flour, probably related to higher humidity in these samples. In contrast, Salisu et al. [38] found fungal contamination with the fungus *A. fumigatus* in maize, rice, and wheat samples, but at a low frequency (1.74%), and Tabuc et al. [39] also found low contamination with *A. fumigatus* of 7.4% in corn samples from Romania.

The presence of *A. fumigatus* has also been observed in other cereals such as rice and wheat, the second most important cereal crop after maize. Thus, Reddy et al. [40] found *A. fumigatus* in rice samples after establishing the contamination of 65 samples from five South Asian countries, finding the presence of five *Aspergillus* spp., with a contamination frequency of 7.27% for *A. fumigatus*. In another study on rice conducted by Douksouna et al. [41] on 98 samples marketed in Kenya (imported from four Asian countries), they found *Aspergillus* spp. in all samples, with the main percentage of occurrence for *Aspergillus flavus* (37.5%), followed by *Aspergillus parasiticus* (27.9%) and *A. fumigatus* (10.8%); contamination with this type of fungi was associated with the high humidity found in the samples (>14%). Regarding fungal contamination in wheat, a study conducted in Romania in 35 samples of wheat and barley showed a high contamination frequency of 77% and 52%, respectively, with the main contaminating species being *A. flavus* and *A. fumigatus* [39]. Meanwhile, in Pakistan, *A. fumigatus* contamination was found in 3.33% of wheat (67 samples) and 16.67% of wheat bran (17 samples) [42].

Beyond raw grains, contamination by *A. fumigatus* has also been reported in processed cereal-based products. Yassein et al. [43] examined the presence of fungi in sixty samples of baby foods, including two cereal products (cerealac and cornflakes); they found that the primary fungal contaminants present in the samples were from the genera *Aspergillus* and *Penicillium*; *A. fumigatus* was present in cerealac samples with a frequency of 3.62% and cornflakes with 0.74%. Meanwhile, Saeed et al. [44] evaluated commercial products from Pakistan with wheat flour (biscuits, pizza, and patties); among the contaminating fungi found *A. fumigatus*, which was present in all three types of samples, associating the presence of fungi to cross-contamination from raw materials, water, containers and handlers to the food. The presence of *A. fumigatus* was also found in a fermented product by Adekoya et al. [45], who evaluated fungal contamination in opaque corn beer in South Africa and found that *A. fumigatus* was present in 11% of the samples; additionally, they established that the beer was contaminated after it was processed, either by contaminated equipment or by the subsequent addition of contaminated ingredients.

The presence of *A. fumigatus* in cereals may pose an inhalation risk for workers during handling and processing. Additionally, contamination in stored grains and derived flour products could lead to unintentional exposure in consumers, particularly those with respiratory conditions [93,94].

#### 2.1.2. Spices

Pepper (*Piper nigrum* L.) is a spice known for its role as a flavoring ingredient. The leading producers of this spice are the countries of Vietnam, Indonesia, India, Brazil, and China; in climatic conditions where high temperature and humidity prevail, this promotes the proliferation of fungi along the production chain [46,95].

Studies on pepper have focused on the search for mycotoxin-producing fungi such as *A. flavus* and *A. parasiticus*; however, other fungi such as *A. fumigatus* have also been found in this search, thus, a study that sought to characterize the toxigenic fungi present in pepper (black and white) from Sri Lanka found a greater presence of fungi in black pepper (10^2^–10^4^ CFU/g) than in white pepper (<10^2^ CFU/g); the dominant fungi were *A. flavus* and *A. parasiticus*, contaminating more than 64% of the samples; other fungi of the genus were also found with an average of 1.48 × 10^2^ CFU/g, among these *A. fumigatus*. All the evaluated samples presented a humidity above 10% and an a_w_ > 0.6 [46]. In a similar study conducted in Romania by Man et al. [47], fungal contamination was evaluated in 35 samples of white and black pepper from 12 commercial establishments, finding for both types of samples >10^3^ CFU/g; the most common was *Aspergillus* spp. (92.6%) and the predominant species *A. fumigatus*, *A. niger*, and *A. flavus*. Gatti et al. [48] evaluated 115 samples of dried black pepper seeds from two producing regions of Brazil, finding an incidence of contamination with filamentous fungi greater than 99% with *Aspergillus* spp. being the predominant genus (53.5%), and 25 strains of *A. fumigatus* were found with an appearance frequency of 6.54%.

The presence of *A. fumigatus* has also been evidenced in spices different from black and white pepper. Thus, a study carried out in Tanzania, where fungal contamination was evaluated in 16 spices from local markets, found *A. fumigatus* in caraway (*Carum carvi*), fenugreek (*Trigonella foenum-graecum*), ginger (*Zingiber offficinale*), and red chilli (*Capsicum annuum*), the high mycological contamination found in some spices such as red chilli and ginger, were attributed to the stage of solar drying and storage [77]. Meanwhile, Hashem and Alamri [49] evaluated 15 spices from Saudi Arabian supermarkets, isolating a total of 520 fungi predominantly of the genera *Aspergillus*, *Penicillium*, and *Rhizopus*; the fungal species *A. fumigatus* was found in two of the spices: fenugreek (*Trigonella foenum-graecum*) and sweet cumin (*Foeniculum vulgare* Mill.) with a contamination frequency of 10 and 20%, respectively.

#### 2.1.3. Coffee (Coffea)

According to the International Coffee Organization [96], coffee production in 2019/2020 cycle was 165 million coffee bags; among the leading producers are Brazil, Vietnam, and Colombia, being one of the most traded products in the world. Due to the coffee production process, there is a risk of fungal contamination at different stages [32]. The presence *A. fumigatus* in different types of samples, varieties, and origins has been evidenced in several studies.

Fungal contamination in coffee has been widely studied, particularly during post-harvest processing. Suárez-Quiroz et al. [52] evaluated fungal contamination during the production of green coffee by the wet, mechanical, and dry processes, finding 80%, 72%, and 90% of fungal contamination, respectively; however, it was found that the process between cherry coffee and green coffee progresses, contamination decreases, which is attributed to the presence of yeasts, bacteria, and other fungi that are better adapted to humid conditions. Regarding *A. fumigatus*, the authors observed the presence in both parchment coffee and green coffee for the wet and mechanical processes, but it was not isolated in samples from the dry stage. Kouadio et al. [50] isolated and identified the fungi present on coffee cherries of the genus *Coffea canephora* in samples from Côte d’Ivoire, finding species such as *A. niger* (40.8–57.2%), *Aspergillus carbonarius* (5.1–32.2%), *Aspergillus ochraceus* (0.05–8.5%), *A. fumigatus* (8.0–27.0%), *A. flavus* (2.5–9.0%), and *Aspergillus japonicus* (1.3–4.6%), some of them related to ochratoxin formation. Culliao and Barcelo [51] evaluated a total of 210 samples from 42 sampling sites of green coffee and coffee cherries of *Coffea arabica* and *Coffea canephora* var. *robusta* in the Philippines, finding fungal contamination superior to 88%, which is attributed to the type of processing used (wet); the genera reported were *Aspergillus*, *Fusarium*, *Mucor*, *Penicillium*, *Rhizopus*, and *Cylindrocarpon*. The authors observed the presence of *A. fumigatus* among the isolates, which were found only in post-harvest samples, mainly in a temperate climate (<20 °C). Another study in the same country was conducted on samples of green coffee from several varieties (*Coffea arabica*, *Coffea canephora* var. *robusta*, *Coffea liberica*, and *Coffea excelsea*) harvested in five provinces from the Philippines; *Aspergillus* spp. contamination was found in 59% of the samples; among these, 11 species were found, with *A. fumigatus* being one of them [53]. In another study, Viegas et al. [32] evaluated 28 samples of green coffee beans from different origins (Africa, Central and South America, and Asia) of *Coffea arabica* and *Coffea canephora* var. *robusta*, finding fungal contamination related to *Aspergillus* spp. in 77.2% of the samples (microbiological culture-based methods) and 96.4% using molecular techniques; eight different species were isolated (*A. candidus*, *A. sydowii*, *A. niger*, *A. ochraceus*, *A. parasiticus*, *A. fumigatus*, *A. flavus*, and *A. versicolor*). Recently, different coffee samples (dried cherries, husk coffee, and green coffee) were collected in the central, east, and west zones of Cameroon from wet and dry processing; fungi of the genera *Mucor*, *Wallemia*, *Acremonium*, *Fusarium*, *Cladosporium*, *Penicillium*, and *Aspergillus* were found to be contaminants; *A. fumigatus* and *A. niger*, *A. carbonarius*, *A. ochraceus*, and *A. nomius* were isolated; in addition, a specific fungal contamination profile was found according to the region of origin [54].

Studies on coffee have focused on evaluating fungal contamination during the post-harvest processes, focusing on ochratoxigenic fungi; only one study has reported the contamination of *A. fumigatus* in processed commercial samples. Casas-Junco et al. [55] evaluated 14 roasted coffee samples and found the predominant genera as *Aspergillus* (95.43%) and *Penicillium* (4.54%), with the *Aspergillus* spp. identified as *A. ochraceus*, *A. carbonarius*, *A. niger*, and *A. fumigatus* (4.54%).

The presence of *A. fumigatus* in coffee beans during the post-harvest and processing stage suggests that fungal spores may persist through drying and storage. Although brewing at high temperatures may reduce fungal viability, further research is needed to determine whether exposure through coffee consumption presents any health risks.

#### 2.1.4. Cocoa (Theobroma cacao)

According to the International Cocoa Organization (ICCO), more than 4 million tons of cocoa are produced each year, with Côte d’Ivoire and Ghana being the main world producers [97]. The cocoa producers’ technological, economic, and social constraints significantly impact quality, especially at the post-harvest stage, as evidenced by the fungal contamination reported [56,57].

One of the first reports of *A. fumigatus* in cocoa was made in 1981 by Niles [58] in beans from nine countries destined to manufacture chocolate, finding colonies of the fungi between 1.1 × 10^1^ CFU/g and 9.0 × 10^5^ CFU/g, being the sample from Granada the one with the highest contamination. In a study conducted in Cameroon by Mounjouenpou et al. [59], the effect of various fermentation methods and their incidence on fungal contamination, especially on ochratoxin-forming fungi, was compared; the main filamentous fungi found were *A. fumigatus*, *A. tamarii*, *A. versicolor*, *A. carbonarius*, *A. niger*, *Penicillium sclerotiorum*, *P. paneum*, *P. crustosum*, *Mucor* spp., *Rhizopus* spp., *Fusarium* spp., and *Trichoderma* spp. The fungus *A. fumigatus* predominated in the samples after fermentation and solar drying. In another study developed in Spain, with fermented and sun-dried cocoa beans from Sierra Leone, Equatorial Guinea, and Ecuador, they found that the predominant fungal *Aspergillus* spp., especially from section *Flavi* and *Nigri*; and strains from *A. fumigatus*, *A. nidulans*, *A. ochraceus*, *A. terreus* and *A. versicolor* species were identified [60]. Copetti et al. [61] analyzed a total of 492 samples of cocoa from different stages (primary stage at the farm, processing, and chocolate making), finding that the highest quantity and diversity of fungi were found in the samples collected at the farm, especially during drying and storage; in the case of *A. fumigatus* it was found only in samples obtained at the primary stage, and the highest frequency of occurrence was during solar drying with 11.76%, followed by fermentation with 3.92%.

#### 2.1.5. Pu-Erh Tea and Yerba Mate

Pu-erh tea is a fermented beverage, especially in Southeast Asia; however, the presence *Aspergillus* spp. dominating the fermentation process can represent a health risk [62]. This has been demonstrated in studies such as that of Wang et al. [63], which evaluated ten microbial isolates of six species recovered from solid-state fermentation of a type of Pu-erh tea, finding the fungal species *Aspergillus tubingensis*, *Aspergillus marvanovae*, and *A. fumigatus*. Another study by Haas et al. [64] examined 36 samples of Pu-erh tea for filamentous fungi, identifying 19 genera and 31 species, with the most common being *Aspergillus acidus* and *A. fumigatus*. In a study by Zhao et al. [65], the presence of fungi in 60 samples was evaluated, finding 62 fungal isolates of 41 species of 19 genera, including 13 species of *Aspergillus* (including *A. fumigatus*) and seven species of *Penicillium*. A similar study was carried out by Zhang et al. [66], where thermophilic fungi in the fermentation process of Pu-erh tea piles were characterized; it was found that high temperatures and low pH provide optimal conditions for the proliferation of fungi, particularly for the species *Blastobotrys adeninivorans*, *Thermomyces lanuginosus*, *Rasamsonia emersonii*, *A. fumigatus*, *Rhizomucor pusillus*, *Rasamsonia byssochlamydoides*, *Rasamsonia cylindrospora*, *A. tubingensis*, *A. niger*, *Candida tropicalis*, and *Fusarium graminearum*.

Yerba Mate (*Ilex paraguariensis*) is a plant produced and highly consumed (dried leaves) in countries such as Brazil, Argentina, Paraguay, and Uruguay; it is usually consumed in infusions with hot or cold water; its stimulant properties (phenolic compounds), antioxidant content and antilipemic activity are known [67]. Regarding fungal contamination, Castrillo et al. [68] evaluated 56 samples of ground yerba mate from different commercial brands and production origins, finding an 80% frequency of *Aspergillus* spp. with the presence of *A. fumigatus* between 6 and 9% of the samples. Arrúa Alvarenga et al. [69] evaluated three samples of compound yerba mate (yerba mate and medicinal herbs) marketed in Paraguay, isolated in different culture media, finding contamination of *Aspergillus* section *Fumigati* in the three samples evaluated. Di Pentima et al. [70] reported the presence of *A. fumigatus* in three of six samples evaluated (leaves and tea bags) sold in the United States (USA); of these samples, five were harvested and packaged in Argentina, and one harvested in Brazil but packaged in the USA. In another work with Yerba Mate, Vieira et al. [98] evaluated the presence of fungal contamination in eight commercial brands from Brazil and the effect of pH 1.5 (gastric pH conditions) and temperature in the infusion preparation; the results showed fungal contamination in the samples, with *A. niger* and *A. fumigatus* being the fungi most frequently present also showing growth at pH 1.5 and high temperatures (>50 °C). Both Di Pentima et al. [70] and Vieira et al. [98] indicate the potential risk of acquiring an invasive fungal disease from the consumption of Yerba Mate in immunocompromised patients. Given that *A. fumigatus* has been identified in Yerba mate and fermented and dried tea leaves, its survival through processing and potential consumption in brewed beverages warrant further investigation, particularly for individuals with underlying respiratory conditions.

#### 2.1.6. Legumes

Legumes, including peanuts, chickpeas, and soybeans, are also prone to *A. fumigatus* contamination. Inadequate storage infrastructure in certain regions may create conditions favorable for fungal proliferation. The ability of *A. fumigatus* to survive in low-moisture environments raises concerns regarding the potential inhalation of spores from dried legume products.

In an evaluation of 95 food samples, including soybeans (*Glycine max* Merr), beans (*Phaseolus vulgaris* L), and peanuts (*Arachis hypogaea* L); the fungal contamination of these samples was determined, 96% of which corresponded to the genera *Aspergillus* and *Penicillium*, also showing the presence of *A. fumigatus* in peanuts and beans, probably due to the high humidity conditions at which peanuts and beans are harvested, unlike soybeans that did not show the presence of *A. fumigatus*, which are harvested under low humidity conditions [34].

In another study carried out in Malaysia, where the contamination of peanuts was evaluated, it was found that the primary fungal contaminant corresponded to *Aspergillus* spp., where *A. fumigatus* was present with a frequency of 1.74% [38]. Meanwhile, in Egypt, 40 peanut samples from 21 different regions showed a contamination frequency of 70% of *A. fumigatus* [71]. In a later study, also carried out in this country, untreated peanuts, roasted peanuts, and roasted and salted peanuts were evaluated; *A. fumigatus* was found in a proportion of 0.01% in untreated peanuts, 0.3% in roasted peanuts and 2.6% in roasted and salted peanuts; also find that contamination with *A. fumigatus* increased with storage time, being higher at 12 and 24 months after storage [72]. Similar findings were made by Kazemi et al. [73] when they examined peanuts, discovering a 12% contamination of *A. fumigatus* in salt-roasted peanuts, which may be connected to post-harvest practices and climatic conditions. Lima et al. [74] evaluated 44 samples of peanuts and peanut products typical of Brazil (“pé de moleque” and “paçoca”), which were purchased in commercial establishments, finding contamination of 11.54% of *A. fumigatus* in the samples. In another study, the phylogenetic diversity and prevalence of fungi in samples of peanuts from street markets and supermarkets in Mafikeng City (South Africa) was evaluated, finding a greater richness of phylotypes in the samples from supermarkets explained by possible variables such as the time and temperature of storage and the type of packaging used. As for *A. fumigatus*, a high incidence (over 70%) was found for both samples [75].

Chickpeas (*Cicer arietinum*) have also been identified as a substrate for fungal contamination. According to Ramirez et al. [76] the fungal genera *Aspergillus*, *Fusarium*, *Penicillium*, *Alternaria*, and *Rhizopus* are the main fungal contaminants isolated in chickpea grains; this was also observed by Shamsi and Khatun [77] in nine varieties of Chickpea, finding contamination of nine species of fungi, during storage; the fungus *A. fumigatus* was found in six of the nine varieties at the beginning of the evaluation period with a frequency between 7% and 28%, at the end of the evaluation (five months), all varieties showed contamination with a frequency between 19% and 46%, increasing with storage time. In another study developed in India by Kumar et al. [78], twenty samples of chickpeas from farmer stores in the Panchgaon region were evaluated; the authors found fungal contamination with 16 species of fungi and an incidence of *A. fumigatus* in the samples between 10% and 20%, depending on the isolation method evaluated.

High humidity storage conditions promote fungal growth in legumes, raising concerns about potential exposure to *A. fumigatus* spores. This contamination risk could be higher in regions with inadequate storage infrastructure, potentially increasing foodborne exposure risks [33].

#### 2.1.7. Nuts and Edible Seeds

In a study by Kazemi et al. [73], untreated, roasted, and salted pistachios and untreated walnuts were evaluated; the authors found that *Aspergillus* spp. had the highest prevalence of contamination in the samples, with 35% for untreated samples and 54.3% for treated samples; in the case of *A. fumigatus* was found it 5% of pistachio samples, 14% in roasted and salted pistachio, and 3% in walnuts; the contamination levels of the products are justified by the authors, due to the temperature and storage conditions. In another study with nuts (almonds, pistachios) and edible seeds, an incidence of *A. fumigatus* of 36.5% in pumpkin seeds, 33.3% in pine nuts, 50% in sunflower seeds, 100% in almonds, and 100% in pistachios was found [79]. There is also a report of *A. fumigatus* contamination in cashew nuts from Lagos (Nigeria), finding up to 50% prevalence of the fungus in this type of nut [80]. In another study in Slovakia, *A. fumigatus* contamination in chestnuts was reported to be 16.5%, and contamination in leaves (12.4%), bark (12.7%), and pollen (12.7%) were also evaluated, showing the ubiquity of this type of fungi [81]. Finally, in a study by Freire et al. [82] on the fungal contamination of Brazil nuts from the Brazilian state of Pará, they found that 4.5% of the samples contained *A. fumigatus*. This contamination was linked to a potential quality issue during drying, processing, and storage. Due to their long shelf life and susceptibility to fungal contamination during storage, nuts and seeds could act as reservoirs for *A. fumigatus* spores. Prolonged storage in inadequate conditions may increase fungal proliferation, posing potential exposure risks, particularly for immunocompromised individuals [33].

#### 2.1.8. Fruits and Vegetables

Adebanjo et al. [83] evaluated the fungal contamination associated with four vegetables (*Abelmoschus esculentus*, *Corchorus olitorius*, *Solanum macrocarpon*, and *Capsicum annum* L.) collected in three markets in Nigeria. The fresh vegetables were subjected to solar drying for five days and subsequently stored for eight weeks. The authors observed that the main fungi present in the samples were *Aspergillus* spp., *Rhizopus* spp., and *Fusarium* spp.; *A. fumigatus* was present in 13.3% of bell pepper (*Capsicum annum*) samples, and in all samples of dried and stored vegetables; they concluded that the isolated fungi mainly were field-acquired and that they managed to survive drying and storage. The prevalence of fungal contamination in processed vegetables was assessed in 30 samples from seven Korean provinces, and results showed that *Aspergillus* spp. had the highest prevalence (80%), with *A. fumigatus* showing up at an average level of 5.0 CFU/g [84].

As for fresh fruits, Serra et al. [84] have reported the presence of five isolates of *A. fumigatus* in grapes at three stages of development from several vineyards in Portugal. Sardiñas et al. [85] also found two isolates of *A. fumigatus* in grapes from the cities of Zamora and Valladolid in Spain. Regarding processed fruit products, Santos et al. [86] evaluated the presence of heat-resistance molds in strawberry puree, orange concentrate, and apple puree subjected to thermal processes, detecting the contamination of this type of fungi in 59.3% of the samples analyzed, finding the teleomorphic phase of *A. fumigatus*, which is called *Neosartorya fumigate* in all samples and the processing stages evaluated; the results showed that the raw material could significantly affect the microbiological quality and stability of the products evaluated. In another study, also in processed fruit, a total of six fungal samples were isolated from pulp samples of apples, strawberries, and oranges; all the fungi isolates were identified as *A. fumigatus* [87].

Regarding health risks, fruits and vegetables, particularly those subjected to drying or prolonged storage, may serve as potential vehicles for *A. fumigatus* spores. While direct foodborne transmission remains unclear, exposure through inhalation during handling and processing should be considered [99,100].

#### 2.1.9. Other Foods

*A. fumigatus* was detected in chicken nuggets (*n* = 7) produced in Brazil; these samples corresponded to customer complaints of chicken nuggets that had not yet expired and had the presence of visible fungi; the results showed that *A. fumigatus* was present in one of the samples (at 5 °C and 25 °C); the authors attribute the *Aspergillus* spp. contamination of the wheat and corn flour [88]. Pena et al. [89] sampled raw cow’s milk in 44 dairy farms in the province of Cordoba in Argentina, finding 45 isolates of *A. fumigatus*; the contamination could be related to environmental contamination of the dairy herd and subsequent contamination of the mammary glands of the cows.

## 3. Possible Pathways of *Aspergillus fumigatus* Contamination in the Food Chain: From Production to Consumption

The main characteristic of *A. fumigatus* is its thermophilic nature, with a minimum growth temperature of 12.8 °C and a maximum between 40 °C and 42.8 °C; it is considered a marginal xerophile due to its minimum growth of 0.82 of water activity (a_w_), and it quickly grows in two days in a petri dish at 37 °C [4]. These characteristics, especially its thermophilic nature, make it possible to find it in different habitats like soil, plant debris, and decomposing organic matter, water-related environments, as well as in the raw materials and stages of food production [4,5,101,102].

**Figure 2:**
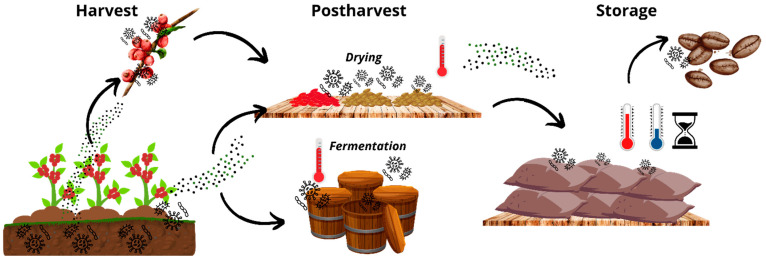
Potential routes of *A. fumigatus* contamination in the food production chain, from environmental exposure to post-harvest handling and storage. Arrows indicate contamination flow along the production chain, from field to consumer.

Considering the above, the first path of contamination towards the food is through the soil, plants, and plant debris through direct contact with the food or the dispersion of spores to the plant, contaminating the food in the field or later stages (Figure 2); this was observed by Freire et al. [82] who found various fungal species from the soil and field contaminating black pepper in the post-harvest stage, with a relationship between temperature and humidity. Pena et al. [89] also found 45 isolates of *A. fumigatus* in raw cow’s milk from 44 dairy farms in Cordoba, Argentina, linking contamination to the contaminated environment of dairy herds. This first path of contamination is linked to the fact that *A. fumigatus* contamination often occurs on food surfaces, as observed in black and white pepper after the removal of the pericarp, with lower fungal contamination [46,47,90].

In the post-harvest phase of food production, contamination by *A. fumigatus* can occur both during crop growth in the field and later in the drying, fermentation, and storage processes and can be related to surrounding fields or cross-contamination as shown in Figure 2 [82]. The growth of fungi, such as *A. fumigatus*, is also influenced by a_w_ and high temperatures during the dehydration stage [4,61]. Gatti et al. [48] have found that *Aspergillus* spp. is predominant in dehydrated pepper seed samples, with *A. fumigatus* present in 6.54% of the samples. Nganou et al. [54] observed a high diversity of fungi after coffee drying, including *A. fumigatus*, implicating air and soil as sources of contamination. Culliao and Barcelo [51] evaluated fungal contamination in green coffee and coffee cherries of *Coffea arabica* and *Coffea canephora* var. *robusta* in the Philippines, finding 75% contamination at harvest and 100% contamination at post-harvest due to the solar drying stage, with *A. fumigatus* growing better in warm climates. Mounjouenpou et al. [59] compared the effect of different fermentation methods in cocoa processing on fungal contamination *A. fumigatus* was one of the dominant fungi in solar-dried samples.

Another environment that provides favorable conditions for *A. fumigatus* is the fermentation stage (Figure 2) because this thermophilic fungus can grow at temperatures above 40 °C [91]. This was observed in Pu-erh tea, a fermented beverage popular in Southeast Asia, but the presence of *Aspergillus* spp. during the fermentation process can pose a health risk because high temperatures and low pH conditions are optimal for the proliferation of *A. fumigatus* [62,66]. A study of Cameroonian cocoa found that most *A. fumigatus* isolates came from samples that had undergone fermentation and drying; filamentous fungi tend to multiply after fermentation [59]. These findings are consistent with the results of Copetti et al. [61], who investigated fungal contamination across cocoa production, finding that *A. fumigatus* only occurred in the primary stage, with a frequency of 11.76% in solar drying, 3.92% in fermentation, and 1.54% in storage.

Food storage during post-harvest is a crucial stage for food safety due to its vulnerability to fungal contamination; however, this stage is vulnerable to contamination of *A. fumigatus* related to the field or contaminated food during the harvest or post-harvest stages [46,47,82,90]. Adebanjo et al. [83] conducted a study on the fungal contamination of four types of vegetables collected from Nigerian markets; the vegetables were solar-dried for five days and then stored for eight weeks, and showed that *A. fumigatus* was present in 13.3% of pepper samples, and all dried and stored vegetable samples, concluding that the fungi were mainly from the field and could survive drying and storage. The growth of *A. fumigatus* tends to increase over time as the storage period progresses; this increase is associated with temperature fluctuations and a decrease in a_w_, which creates a favorable environment for the growth of *A. fumigatus* and other similar species [38,61,72,75].

Finally, during the processing stage, it is common to find *A. fumigatus* in ready-to-eat foods, where contamination is most likely to occur during drying or storage, which is typical for foods such as spices, legumes, cereals, nuts, and fruits (Figure 1). However, in foods that have undergone thermal treatments, the presence of this mold is not common, but there is evidence, such as a study on fruit juices and concentrates, where the presence of heat-resistant fungi was evaluated, finding Neosartorya fumigate, the teleomorph phase of *A. fumigatus*; attributing this to the introduction of raw materials into processing plants, suggesting that raw materials could significantly impact the microbiological quality and stability of the product Santos et al. [86]. Casas-Junco et al. [55] evaluated various samples of roasted coffee and found a contamination frequency of 4.54% with *A. fumigatus*; however, the contamination source is unclear. Saeed et al. [44] studied commercial products made from wheat flour in Pakistan (biscuits, pizza, and patties), finding *A. fumigatus* in all three types of samples, which they attributed to cross-contamination from raw materials, water, containers, and handlers.

The widespread presence of *A. fumigatus* in agricultural and food products raises concerns beyond contamination. While some studies have reported an association between environmental exposure to azole fungicides and the emergence of resistant *A. fumigatus* strains, it remains crucial to note that this is an observation rather than a confirmed causal relationship. More comprehensive research is needed to elucidate the transmission dynamics between environmental and clinical isolates and assess the extent to which agricultural practices contribute to resistance development in clinical settings.

## 4. Azole Resistance in *Aspergillus fumigatus*

Azoles are chemicals used to prevent the growth of fungi that cause disease in humans, animals, and plants; these are the primary substances used to treat aspergillosis, a life-threatening condition with mortality rates of 50–100%, due to the limited number of effective antifungals [6,103]. Azole drugs are primarily considered fungistatic agents, as they inhibit ergosterol biosynthesis and prevent fungal growth. Although some studies indicate that azoles may display fungicidal activity, this effect occurs only under specific experimental conditions of prolonged exposure or high concentrations, which significantly exceed the environmental levels typically encountered. Therefore, their primary role remains fungistatic [104,105].

Clinical azoles such as voriconazole (primarily used for invasive infections), isavuconazole (mainly used for allergic and chronic aspergillosis), and posaconazole (used in the immunocompromised host) are the most commonly prescribed antifungals for the treatment of aspergillosis in human patients due to their efficacy and low toxicity [5,101]. However, the rise of azole resistance in both clinical and environmental strains of *A. fumigatus* may have detrimental effects on treating invasive human fungal infections [106]. Clinical strains of *A. fumigatus* from the USA were the first to show signs of azole resistance in the late 1980s, and more recent studies have shown that azole resistance has been on the rise in Europe and other regions since the late 1990s [6,107,108,109]. Azoles disrupt the activity of sterol 14α-demethylase (Cyp51), an enzyme essential for synthesizing sterols needed to maintain the structure of fungal cell membranes [110]. *A. fumigatus* has two forms of Cyp51: Cyp51A and Cyp51B and many strains of this species have developed resistance to azoles due to mutations in Cyp51A [25,108]. Moreover, clinical isolates have demonstrated other resistance mechanisms, such as increased expression of Cyp51, the presence of efflux pumps, accumulation of ergosterol precursors, and reduced intracellular retention of azoles [111,112,113,114,115]. These clinical strains, together with many azole-resistant environmental isolates, can be identified as belonging to two distinct genotypes based on a combination of their Cyp51A alterations and promoter tandem repeat (TR) inserts of 34 or 46 bp [109,116,117]. Clinical isolates containing the Cyp51A inserts TR34 and the amino acid substitution L98H [TR34, L98H] were first found in Europe in 1998, in contrast to the first resistant isolate identified from North America in 2008, which had the Cyp51A insert TR46 and the amino acid alterations Y121F and T289A [109,116,117]. Since 2007, highly azole-resistant isolates have been found in azole-naïve (i.e., untreated) patients in the Netherlands [118]. The genotypes that have become prevalent globally are known for their high resistance to agricultural azoles; they are also commonly used to preserve materials like wood and fabrics, control fungal diseases in animals and crop plants, and prevent post-harvest losses in horticulture [119]. It is noteworthy that the TR34/L98H allele, a key contributor to azole resistance, can be found in *A. fumigatus* isolates from every continent, making up a significant proportion of resistant isolates in environmental settings [5].

**Figure 3:**
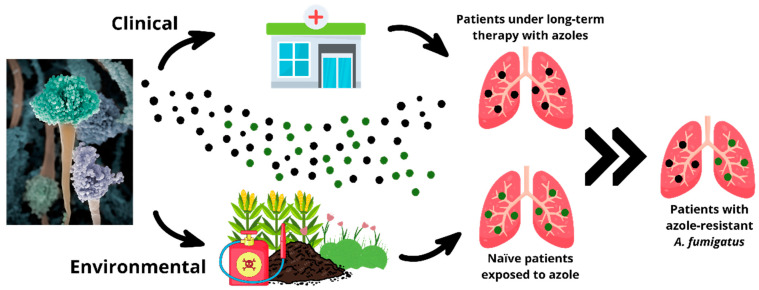
Resistance to azole fungistatic agents in *A. fumigatus* arises through clinical and environmental pathways. The clinical pathway results from prolonged azole treatment in patients, while the environmental pathway involves exposure to azole fungicides in agriculture and veterinary drugs in animal husbandry, both contributing to resistant strains in the environment. Black dots indicate spores from clinical pathway, while green dots represent spores from environmental pathway.

Considering the information presented, azole resistance in *A. fumigatus* can arise through multiple pathways. The two main routes include environmental exposure to azole fungicides and long-term clinical treatment with azole antifungals [15,103,112]. Additionally, recent studies suggest that the livestock industry may also contribute to azole resistance, as azole-based antifungals are sometimes used in animal husbandry, potentially exposing *A. fumigatus* to selective pressure [13,14]. The clinical route is observed in patients who received prolonged treatment with azoles for aspergillosis, and the second resistance is acquired through environmental exposure to azoles, primarily in agricultural environments (Figure 3) [15,119]. There are concerns about the potential for azole fungistatic agents to promote resistance in *A. fumigatus*, a non-target pathogen, through environmental exposure in agroecosystems [15,103,120]. Studies have observed an association between azole applications for veterinary purposes, crop spraying in agriculture, or material preservation and the presence of resistant *A. fumigatus* isolates. Nevertheless, more evidence is needed to confirm whether this reflects a direct causal relationship [121]. This may be linked to the structural similarity between clinical and agricultural azoles, particularly difenoconazole, propiconazole, epoxiconazole, bromuconazole, and tebuconazole, which can result in cross-resistance [122,123]. Moreover, a recent study conducted by the European Food Safety Authority (EFSA) [13] emphasized that between 2010 and 2021, around 99.16% of the triazoles marketed in the European Union were used for crop protection. This suggests a possible association between environmental exposure to triazoles and the selection of cross-resistance to medical azoles in *Aspergillus* spp. Nevertheless, more research is required to establish a direct causal relationship.

As has been evidenced previously, the resistance of *A. fumigatus* to agricultural azole fungistatic agents is an important issue, as this species of fungus is a significant pathogen that can cause severe illnesses in humans. For this reason, various investigations have been conducted to understand the relationship between the resistance of *A. fumigatus* to agricultural azole and the potential causes behind the resistance. Table 1 shows a summary of these studies’ results, where different isolates of *A. fumigatus*, obtained from soil, woody debris, decaying material, and flower waste compost samples, were evaluated and subjected to different concentrations of various agricultural and clinical fungicides. Sensitivity analysis techniques or resistance induction studies were used in samples exposed to these types of azole agents. The detection of azole residues in agricultural environments suggests a possible association with resistance in *A. fumigatus* isolates, though further studies are needed to confirm this relationship.

As shown in Table 1, around 19 types of azole fungicides used in agriculture have been evaluated, mainly triazoles, the most studied being tebuconazole, with a frequency of 15%. The most evaluated samples were soils from different origins, including hospital parking lots, gardens, trees, wood, greenhouses, and tulip fields, as well as decaying plant material and clinical strains, mainly used in resistance induction studies with agricultural fungicides [18,19,20,22,24]. The reported minimum inhibitory concentrations (MIC) ranged from 0.125 mg/L to 43.153 mg/L, with the highest values found for the fungicide Imazalil in the Netherlands and the United Kingdom [24,25]. The most reported resistance changes in these studies include TR34/L98A, TR46/Y121F/T298A, and TR34/L98H/S297T/F495I, which are associated with mutations in Cyp51A.

## 5. Future Perspectives on the Evaluation and Control of *A. fumigatus* in Food and Consumer Health Risks

*A. fumigatus* is a fungus commonly found in food that can be potentially dangerous to human health. This fungus has been associated with resistance to azoles and is also one of the main causes of aspergillosis [5,106]. The current presence of *Aspergillus* spp. resistant to azoles in food and food production environments poses a risk to consumers and workers in the agri-food industry, as they can acquire resistance through the “environmental pathway” [30,31]. This risk is even more latent following the COVID-19 pandemic and the WHO statement that *A. fumigatus* is one of the fungi with the greatest threat to human health [9,11]. For this reason, there is an urgent need to carry out assessments to determine the risk of the presence of azole-resistant *A. fumigatus* and its presence in food, taking into account that this fungus is a common contaminant of foods such as cereals, nuts, spices, legumes, coffee, cocoa, tea, and some fruits, and may therefore be an additional route for the acquisition of environmental resistance to azoles.

The recent evidence of *Aspergillus* section *Fumigati* in food and its resistance to azole fungistatic agents represent a growing threat to public health [30]. This situation requires immediate attention to fill some of the knowledge gaps regarding the relationship between food, *A. fumigatus*, and the potential health risks. A key challenge in assessing *A. fumigatus* in food is the lack of standardized environmental baseline data. Unlike mycotoxin-producing fungi with regulatory limits, no reference values exist to distinguish natural presence from contamination. Future research should focus on establishing these baselines to improve risk assessment and regulatory decisions.

Future studies should also investigate whether exposure to *A. fumigatus* through food contributes to human colonization or infection, particularly in high-risk populations. Additionally, it is crucial to determine whether the ingestion of contaminated food could lead to fungal persistence in the gastrointestinal tract, contribute to gut microbiota imbalances, or act as a secondary source of exposure for immunocompromised individuals [124]. Understanding the viability and pathogenic potential of ingested fungal spores is essential to determine whether this represents an additional route for acquiring aspergillosis or azole-resistant strains.

In this sense, studies should be conducted to determine the prevalence of azoles in agricultural environments and post-harvest food. Similarly, it is necessary to identify the most susceptible environments for the growth of *A. fumigatus* at this stage, to validate the routes of contamination of the fungus from the field to the table, to investigate the relationship between the “hotspots” identified in studies of azole resistance in agriculture and the fermentation processes of some agricultural products intended for human consumption [5,125], to increase knowledge of the residues of azole compounds in food, to identify the types of mutations present in isolates of *A. fumigatus* isolates resistant to azoles in food and the impact of climate change on the growth of this fungus. Further research is essential to identify the factors driving azole resistance in *A. fumigatus*. Studies should focus on how resistance develops in the environment and its impact on the food production chain, addressing key questions such as “where”, “how”, “at what stage”, and “how often” it occurs. This approach could help pinpoint high-risk scenarios for resistance selection across different azole application areas.

It is also important to implement intervention actions to reduce the risk of the presence of *A. fumigatus* in food and its resistance to azoles. This implies educating and raising awareness among the agri-food chain actors about the responsible use of fungicides, resistance to them, the effects of *A. fumigatus* on health, and the resulting impacts. In addition, it is also important to closely monitor the emergence of azole-resistant *A. fumigatus*, research and create rapid diagnostic methods for field application, and use physical, chemical, and biological control methods to control *Aspergillus* spp. in post-harvest stages; this is especially important in post-harvest stages such as drying, fermentation, and storage.

## 6. Conclusions

In this review, we have summarized the occurrence of *Aspergillus fumigatus* in agricultural and food products, its potential role in azole resistance, and the implications for public health. Although studies have reported an association between environmental exposure to azole fungistatic agents in agricultural settings and the presence of resistant strains, further research is required to confirm this association and establish any causal link.

However, it is important to note that the detection of *A. fumigatus* in food does not necessarily indicate the presence of azole-resistant strains, as resistance requires specific testing. Furthermore, the absence of standardized environmental baseline data makes it difficult to distinguish between natural fungal presence and actual contamination, requiring intervention.

Further research is essential to assess whether the ingestion of contaminated food contributes to human colonization or infection, particularly in vulnerable populations. Strengthening surveillance, promoting responsible fungicide use, and improving diagnostic methods will be key to mitigating the risks associated with *A. fumigatus* in the food production chain. Moreover, variations in temperature and humidity driven by climate change may further influence the presence and persistence of *A. fumigatus* in agricultural environments and food products, potentially exacerbating the risk of contamination and resistance selection.

Addressing these challenges requires a multidisciplinary approach, integrating microbiology, food safety, and public health strategies to minimize the impact of azole-resistant *A. fumigatus* on consumers and the environment.

## Figures and Tables

**Table 1:** Recent research in azole resistance detected in agricultural and environmental samples of *Aspergillus fumigatus*.

Samples	Origin of Isolates	Number of *A. fumigatus* Isolates	Type of Fungicides Azoles Evaluated	Concentration of Azoles Fungicides (mg/L)	MIC (mg/L) *	CYP51A Aminoacid Substitutions Associated with Azole Resistance	References
Soil and woody debris	Tanzania	15	itraconazole	0.03 to 16	1 to 2	TR34/L98A and TR46/Y121F/T298A	[18]
			voriconazole	0.03 to 16	>16		
			posaconazole	0.015 to 8	0.25 to 0.5		
			isavuconazole	0.015 to 8	8		
			bromuconazole	0.06 to 32	>32		
			cyproconazole	0.06 to 32	>32		
			difenoconazole	0.06 to 32	>32		
			epoxiconazole	0.06 to 32	>32		
			penconazole	0.06 to 32	>32		
			tebuconazole	0.06 to 32	>32		
			triadimefon	0.06 to 32	>32		
			metconazole	0.06 to 32	>32		
			hexaconazole	0.06 to 32	>32		
			tricyclazole	0.06 to 32	>32		
pumpkin farm (air samples from agricultural field)	Japan	50	itraconazole	0.19 to 1.5	0.74	NR	[19]
			posaconazole	0.19 to 1.5	0.006 to 0.16		
			voriconazole	0.19 to 1.5	0.47 to 0.64		
			tetraconazole	580 to 1132	≤36.25		
Soil samples from greenhouses for vegetables and fruits	China	73	itraconazole	NR	0.5 to 16	TR46/Y121F/T289A; A284T; G448S; P222Q and TR34/L98H/S297T/F495I	[20]
			posaconazole	NR	0.25 to 1		
			voriconazole	NR	1 to 16		
			epoxiconazole	1 to 16	NR		
			tebuconazole	1 to 16	NR		
			propiconazole	1 to 16	NR		
			hexaconazole	1 to 16	NR		
			metconazole	1 to 16	NR		
Environmental isolates	China	24	itraconazole	NR	0.25 to ≥16	TR34/L98H/S297T/F495I; G54R; G54V and TR46/Y121F/T289A	[21]
			voriconazole	NR	0.25 to ≥16		
			posaconazole	NR	0.125 to ≥8		
			epoxiconazole	0.06 to 32	0.5 to ≥32		
			bromuconazole	0.06 to 32	0.25 to ≥32		
			tebuconazole	0.06 to 32	0.5 to ≥32		
			difenoconazole	0.06 to 32	0.25 to ≥32		
			propiconazole	0.06 to 32	1 to ≥32		
			imazalil	0.06 to 32	0.06 to ≥32		
			prochloraz	0.06 to 32	0.125 to ≥32		
Soil samples	China	2	itraconazole	NR	0.5 to 16	TR46/Y121F/T289A	[22]
			voriconazole	NR	0.25 to >16		
			posaconazole	NR	0.125 to 0.5		
			tebuconazole	0.5 to 5	0.5 to >16		
Soil samples	China	21	itraconazole	4	1 to >16	TR34/L98H; TR34/L98H/S297T/F495I and TR46/Y121F/T289A	[23]
			voriconazole	2	1 to >16		
			posaconazole	0.5	1 to 2		
			epoxiconazole	0.06 to 32	2 to >32		
			bromuconazole	0.06 to 32	1 to >32		
			tebuconazole	0.06 to 32	2 to >32		
			difenoconazole	0.06 to 32	0.5 to >32		
			propiconazole	0.06 to 32	2 to >32		
			prochloraz	0.06 to 32	0.25 to >32		
			imazalil	0.06 to 32	0.125 to >32		
			voriconazole	0.03 to 32	0.25 to >32		
			isavuconazole	0.03 to 32	0.5 to >32		
			posaconazole	0.03 to 32	0.125 to 2		
			prothioconazole	0.016 to 16	4 to >32		
			paclobutrazole	0.016 to 16	16 to >32		
			epoxiconazole	0.016 to 16	4 to >32		
			propiconazole	0.016 to 16	4 to 32		
			tebuconazole	0.016 to 16	2 to >32		
			difenoconazole	0.016 to 16	1 a >32		
			metconazole	0.016 to 16	0.25 to 16		
			prothiozonacole-desthio	0.016 to 16			
			imazalil	0.016 to 16	0.125 to 32		
			prochloraz	0.016 to 16	0.25 to 32		
			mefentriflfluconazole	0.016 to 16	>32		
Tulip field soils, flower bulbs, tulip peel waste heaps (decaying material), flower waste compost sites, clinical isolates	Netherlands and UK	200	voriconazole	0.1 to 19.120	0.292 to >19.120	TR46/Y121F/T289A; TR34/L98H/T289A/I364V/G448S; F46Y/M172V/E427K; TR34/L98H/S297T/F495I and TR34/L98H	[24]
			imazalil	0.25 to 43.153	0.714 to >43.153		
			tebuconazole	0.1 to 17.349	0.643 to >17.349		
Soil samples and wheat straw	UK	692	voriconazole	0.1 to 19.120	0.342 to >19.120	TR46/Y121F/T289A; TR46/Y121F/M172V/T289A/G448S and TR46/Y121F/T289A/S363P/I364V/G448S	[25]
			itraconazole	0.025 to 22.324	1.056 to >22.234		
			imazalil	0.25 to 43.153	0.978 to >43.153		
			tebuconazole	0.1 to 17.349	0.829 to >17.349		
Soil samples	China	162	itraconazole	NR	1 to >16	TR34/L98H and TR34/L98H/S297T/F495I	[26]
			voriconazole	NR	0.5 to 8		
			posaconazole	NR	0.25 to 2		
			tebuconazole	NR	8 to 16		
Soil samples from rural and urban locations	México, Paraguay, Perú, Benin and Nigeria	NR	tebuconazole	NR	0.5 to 32	TR34/L98H and TR46/Y121/T289A	[27]
			itraconazole	4	>16		
			voriconazole	2	1		
Soil, plant debris, and compost	USA	748	tebuconazole	3	1 to >16	TR46/Y121F/T289A; I242V and Y46F/V172M/T248N/E255D/K427E	[28]
			itraconazole	0.015 to 16	0.5 to 2		
			voriconazole	0.015 to 16	0.25 to >16		
			posaconazole	0.015 to 16	0.25 to 1		

* All *A. fumigatus* isolates were obtained from the listed agricultural and environmental samples. Azole resistance in *A. fumigatus* was determined using an MIC threshold of ≥2 mg/L, following reference guidelines of the European Committee on Antimicrobial Susceptibility Testing (EUCAST) [29]. NR: not reported.

## Data Availability

No new data were created or analyzed in this study.
